# Prenatal diagnosis of severe mitochondrial diseases caused by nuclear gene defects: a study in Japan

**DOI:** 10.1038/s41598-021-81015-y

**Published:** 2021-02-11

**Authors:** Nana Akiyama, Masaru Shimura, Taro Yamazaki, Hiroko Harashima, Takuya Fushimi, Tomoko Tsuruoka, Tomohiro Ebihara, Keiko Ichimoto, Ayako Matsunaga, Megumi Saito-Tsuruoka, Yukiko Yatsuka, Yoshihito Kishita, Masakazu Kohda, Akira Namba, Yoshimasa Kamei, Yasushi Okazaki, Shinji Kosugi, Akira Ohtake, Kei Murayama

**Affiliations:** 1 Center for Medical Genetics, Chiba Children’s Hospital, Chiba, Japan; 2 Department of Medical Genetics/Medical Ethics, Kyoto University School of Public Health, Kyoto, Japan; 3 Department of Metabolism, Chiba Children’s Hospital, 579-1 Heta-cho, Midori-ku, Chiba, 266-0007 Japan; 4 Department of Pediatrics, Faculty of Medicine, Saitama Medical University, Saitama, Japan; 5 Department of Neonatology, Chiba Children’s Hospital, Chiba, Japan; 6 Department of Clinical Genomics, Faculty of Medicine, Saitama Medical University, Saitama, Japan; 7 Center for Intractable Diseases, Saitama Medical University Hospital, Saitama, Japan; 8 Diagnostics and Therapeutics of Intractable Diseases, Intractable Disease Research Center, Graduate School of Medicine, Juntendo University, Tokyo, Japan; 9 Department of Life Science, Faculty of Science and Engineering, Kindai University, Osaka, Japan; 10 Department of Obstetrics and Gynecology, Saitama Medical University Hospital, Saitama, Japan

**Keywords:** Metabolic disorders, Clinical genetics

## Abstract

Prenatal diagnoses of mitochondrial diseases caused by defects in nuclear DNA (nDNA) or mitochondrial DNA have been reported in several countries except for Japan. The present study aimed to clarify the status of prenatal genetic diagnosis of mitochondrial diseases caused by nDNA defects in Japan. A comprehensive genomic analysis was performed to diagnose more than 400 patients, of which, 13 families (16 cases) had requested prenatal diagnoses. Eight cases diagnosed with wild type homozygous or heterozygous variants same as either of the heterozygous parents continued the pregnancy and delivered healthy babies. Another eight cases were diagnosed with homozygous, compound heterozygous, or hemizygous variants same as the proband. Of these, seven families chose to terminate the pregnancy, while one decided to continue the pregnancy. Neonatal- or infantile-onset mitochondrial diseases show severe phenotypes and lead to lethality. Therefore, such diseases could be candidates for prenatal diagnosis with careful genetic counseling, and prenatal testing could be a viable option for families.

## Introduction

Mitochondrial diseases have the highest incidence rate (1 in 5000 births) among all hereditary metabolic disorders^1^. Mitochondria are primarily responsible for generating adenosine triphosphate by oxidative phosphorylation. Two distinct genomes can contribute to mitochondrial disease pathogenesis: the nuclear genome and the maternally inherited 16.6 kb mitochondrial genome^2,3^. Mitochondrial diseases result from mutations in either of these genomes. Defects in nuclear DNA (nDNA) can cause problems such as defects in respiratory chain complex structure, translation, and mitochondrial DNA (mtDNA) maintenance and repair^3,4^. Approximately 25% of the mitochondrial diseases diagnosed in childhood are due to mtDNA abnormalities, whereas the remaining 75% are due to nDNA defects^5,6^.

Severe neonatal- or infantile-onset mitochondrial diseases usually result in death within the first year of life^5,6^. A study reported that lethal infantile mitochondrial disease (LIMD) constitutes approximately 8.5% of the cases of childhood-onset mitochondrial diseases^6^. Most cases of LIMD are diagnosed biochemically and genetically after the decease of subjects. Such cases of severe neonatal-onset mitochondrial diseases such as LIMD, Leigh syndrome, and mtDNA depletion syndrome may therefore serve as candidates for prenatal diagnosis when the parents wish to conceive again.

Approximately 20,000 women in Japan undergo invasive prenatal genetic testing every year^7^, and the most relevant reason is advanced maternal age. Prenatal diagnoses of mitochondrial diseases caused by nDNA or mtDNA defects are prevalent in several countries^8–10^, but not in Japan. The Japan Society of Obstetrics and Gynecology has issued guidelines for prenatal diagnosis of severe pediatric-onset diseases caused by chromosomal abnormalities or nDNA defects. However, there have been no guidelines for the diagnosis of mtDNA-associated diseases^11^. Thus, our work focus on the prenatal diagnosis of mitochondrial diseases caused by nDNA defects.

The advent of next-generation sequencing has increased the diagnostic yield for mitochondrial diseases, concomitant with the increasing need for prenatal diagnosis. However, little information is available on genetic counseling for mitochondrial diseases, especially in Japan. Therefore, this study aimed to clarify the status of prenatal genetic diagnosis of mitochondrial diseases caused by nDNA defects in Japan.

## Results

**Figure 1:**
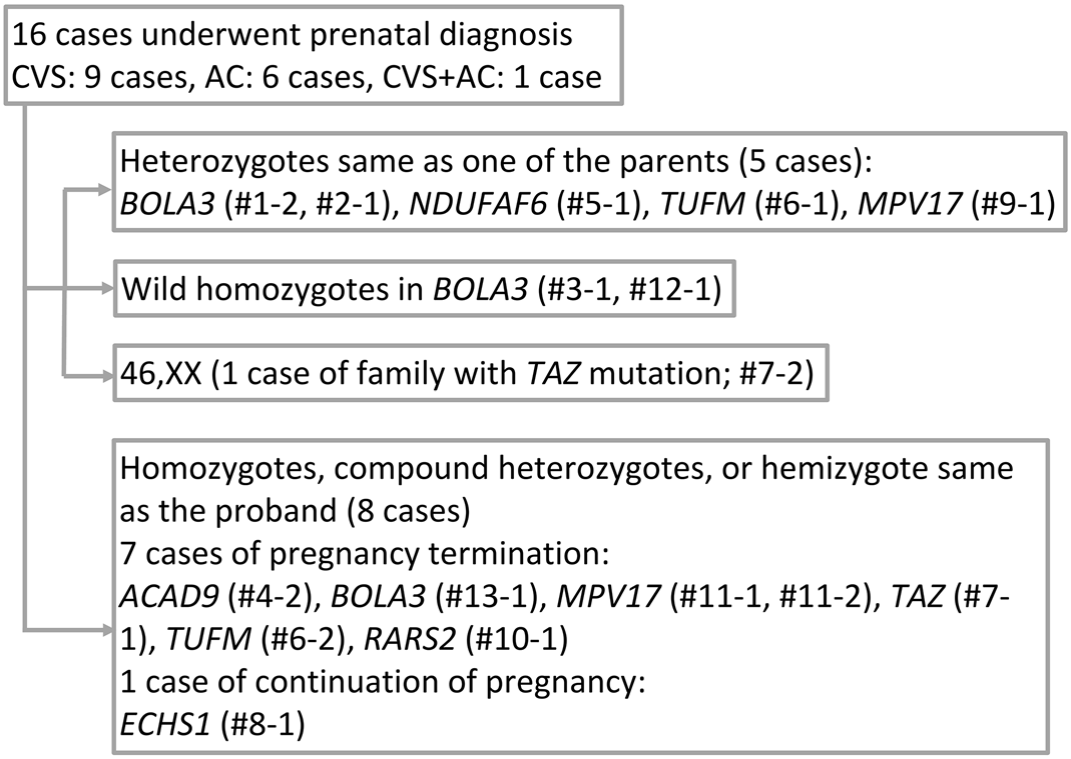
Results of prenatal testing. We conducted prenatal testing for 16 cases. Of them, eight fetuses were not affected, and the pregnancies were continued. The other eight fetuses had the same pathogenic variants as the proband. One family decided to continue the pregnancy and the other seven opted for the termination of pregnancy. *CVS* chorionic villus sampling, *AC* amniocentesis.

In this study, 16 prenatal diagnoses for 13 families were performed safely between August 2014 and January 2020. No complications occurred during the chorionic villus sampling (CVS) and amniocentesis (AC). Figure 1 shows the results of the prenatal diagnosis and details of the pregnancy course: Five cases were found to be carriers of autosomal recessive genes. Two cases were proven as wild type homozygous for *BOLA3*. As the chromosomal analysis in the Family (Fam) #7-2 with the *TAZ* mutation showed the fetus was a female, we did not conduct further genetic analysis. In these eight cases, the parents decided to continue the pregnancy and gave birth to healthy children. The other eight cases were found to carry pathogenic variants in the compound heterozygous or hemizygous states. The parents of Fam #8-1 decided to continue the pregnancy, while those of the remaining seven cases decided to terminate the pregnancies.

## Discussion

In this study, we have shown a summary of prenatal diagnoses for mitochondrial diseases caused by nDNA mutations in Japanese patients. This is the first comprehensive study of prenatal diagnoses for mitochondrial diseases in Japan.

In our cohort, seven probands developed initial symptoms during the neonatal period (< 1 month). The other six probands developed initial symptoms during the infantile period (< 1 year). Moreover, among all cases, seven probands died before completing 1 year of age. Five families had *BOLA3* mutations, and all the probands died. Among them, four (Fam #1, #2, #3, and #12) patients had homozygous c.287A>G, while one from Fam #13 had compound heterozygous c.287A>G with another mutation. Our previous report suggested that this variant is common in the Japanese population and originates from a single founder^14^. Moreover, two probands had *MPV17* mutations (Fam #9 and #11) and developed severe liver manifestations. *MPV17* is the most common gene in hepatocerebral mitochondrial DNA depletion syndrome (MTDPS) in the Japanese population, and c.451dupC in *MPV17* is a frequently occurring mutation^15^. A homozygous c.451dupC mutation has also been identified in Korean siblings that died of liver failure at 6 months of age, and homozygosity is assumed to be associated with poor outcomes^15,16^.

As described in the indexed case profile, in Fam #12, only the mother was a carrier for the pathogenic variant. The large deletion in or around *BOLA3* in his father was absent. *BOLA3*-associated mitochondrial diseases follow an autosomal recessive inheritance pattern. In some cases, only one parent is the carrier, and the mutation is segregated to the patient through uniparental isodisomy (UPiD)^17^. The maternal isodisomy of the whole chromosome 2 was confirmed using short nucleotide polymorphism experiments. Although we explained repeatedly that the recurrence risk is almost null in the case of UPiD, prenatal diagnosis was conducted upon strong requests by the parents.

During prenatal genetic counseling, parents and families consider the multiple options available and make a decision based on the impact of the tests on their lives and their future child in various ways. In our cohort, some families did not opt for prenatal diagnosis after considering the risks of villus and amniotic fluid tests. In addition, one family (Fam #8) decided to give birth to an affected child after prenatal diagnosis. An important role of genetic counselors is to provide accurate information about tests and support the family’s decisions^9,18^. It is an immense challenge for the families to give birth to a child who would follow a severe fate after birth. Therefore, it is necessary to obtain the unified cooperation of obstetricians, neonatologists, pediatricians, and other healthcare providers. The samples for prenatal tests are obtained via CVS or/and AC. CVS and AC are usually performed at 11–14 and after 15 weeks of gestation, respectively. The spontaneous miscarriage rate is approximately 1% and 0.2–0.5% for CVS and AC, respectively^19^. Wulff et al. reported that neither CVS nor AC have any significant effect on the risk of miscarriage or stillbirth^20^. In our cohort, no spontaneous miscarriages occurred as a result of prenatal testing.

Prenatal diagnosis for mtDNA-related mitochondrial diseases is prevalent in several countries^8–10^. Furthermore, preimplantation genetic testing for Leigh syndrome caused by mtDNA mutation has been reported in Japan^21^. However, recent studies indicated that prenatal samples provide an accurate prediction of mtDNA mutation load in the postnatal period^22^. Nonetheless, the relationship between genotype and phenotype is far less clear with regard to some mtDNA mutations. Therefore, further research to ascertain the correlation between genotype, mutation load, and phenotype is required concerning such mtDNA mutations. Moreover, a recent study revealed that de novo mtDNA point mutations are common, and the recurrence risk is low^23^. The Japan Society of Obstetrics and Gynecology guideline does not mention a prenatal diagnosis of mtDNA-associated diseases^11^. Therefore, careful consideration of prenatal testing for mtDNA-associated diseases is needed.

This study provides a comprehensive account of the status of prenatal diagnosis of mitochondrial diseases caused by nuclear gene defects in Japan. As shown in our previous report, neonatal- or infantile-onset mitochondrial diseases exhibit an extremely severe phenotype^5^. Furthermore, there are only a few treatments available for such severe cases. Since the diagnostic ability of genetic tests for neonatal- or infantile-onset mitochondrial diseases is steadily increasing^24^, it is presumed that the demand for prenatal diagnosis of neonatal-onset mitochondrial diseases will also increase. Further discussions and data collection are needed for a more effective application of prenatal testing of mitochondrial diseases caused by both nDNA and mtDNA defects.

## Conclusions

Neonatal- or infantile-onset mitochondrial diseases often show severe phenotype and follow a lethal course. These diseases could be candidates for prenatal diagnosis, and prenatal testing should be considered as one of the important options for parents. Therefore, adequate and appropriate genetic counseling is required before and after prenatal testing.

## Methods

All methods were performed in accordance with the relevant guidelines and regulations.

### Ethics approval and consent to participate

The institutional review board/ethics committee at Saitama Medical University approved the protocol of prenatal diagnosis. All parents provided informed written consent before prenatal testing.

### Consent for publication

Written informed consent was obtained from the parents of all subjects included in the study.

### Molecular and genetic diagnoses of the index case

First, the analyses of enzyme activities and oxygen consumption rates were performed for patients who were suspected to have mitochondrial diseases. Thereafter, whole mtDNA and whole-exome sequencing and validation tests were performed. Genetic counseling, before and after prenatal diagnosis, CVS, and AC, was performed at the Department of Pediatrics and Department of Obstetrics and Gynecology, Saitama Medical University Hospital. The obtained samples were analyzed at Juntendo University.

### Prenatal diagnosis

**Figure 2:**
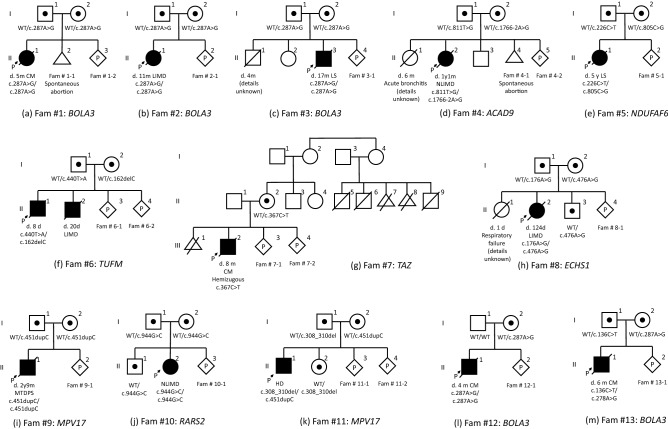
Family tree of 13 probands. One family had X chromosome-linked gene mutation; the other 12 families had autosomal recessive gene mutations. In our cohort, there was no consanguineous couple. *Fam* family, *CM* cardiomyopathy, *LIMD* lethal infantile mitochondrial disease, *NLIMD* non-lethal infantile mitochondrial disease, *LS* Leigh syndrome, *HD* hepatic disease, *MTDPS* mitochondrial DNA depletion syndrome, *WT* wild type, *m* month, *d* day, *y* year.

In total, 13 probands from 13 families were diagnosed with severe mitochondrial diseases caused by pathogenic variants in nDNA. Most of the probands had homozygous or compound heterozygous variants in the autosomal recessive genes [*ACAD9* (MIM 611103) (n = 1)*, BOLA3* (MIM 613183) (n = 5)*, ECHS1* (MIM 602292) (n = 1)*, MPV17* (MIM 137960) (n = 2)*, NDUFAF6* (MIM 612392) (n = 1)*, RARS2* (MIM 611524) (n = 1)*, TUFM* (MIM 602389) (n = 1)]. Only one proband had a hemizygous variant in the X chromosome-linked gene [*TAZ* (MIM 300394) (n = 1)]. Table 1 and the following section describes the details of each proband and family. Figure 2 shows the family tree of each family. The 13 families (18 cases) decided to undergo prenatal diagnosis.

**Table 1:** Clinical characteristics of 13 probands.

Pt no	Family no	Sex	Mutation	Clinical diagnosis	Consanguinity, family history	Initial symptom	Onset	Status	Clinical picture	Affected complex
314	#1	Female	*BOLA3* c.287A>G/c.287A>G	CM	(−)	Vomiting	2 m	Dead (5 m)	Liver failure, lactic acidosis, cardiomyopathy	CI + III + IV
268	#2	Female	*BOLA3* c.287A>G/c.287A>G	LIMD	(−)	Feeding difficulty	24 d	Dead (11 m)	Apnea, epilepsy	CI + II
286	#3	Male	*BOLA3* c.287A>G/c.287A>G	LD + FCMD	(−) EB died at 4 m	Lactic acidosis, hyperglycemia, elevated creatine kinase	17 d	Dead (17 m)	Muscle weakness, hypotonia, cardiomyopathy	CI + II
25	#4	Female	*ACAD9* c.1766-2A>G/c.811T>G	NLIMD	(−) ES died at 6 m	Myotonia	5 m	Dead (1 y 1 m)	Hypertrophic cardiomyopathy, lactic acidosis	CI
512	#5	Female	*NDUFAF6* c.805C>G/c.226T>C	LD	(−)	Regression	7 m	Dead (5 y)	Lactic acidosis	CI
622	#6	Male	*TUFM* c.440T>A/c.162delC	LIMD	Unknown YB died at 20 d	ELBW infant	0 d	Dead (0 m)	Lactic acidosis, apnea	CI + III + IV
1401	#7	Male	*TAZ* c.367C>T	CM	(−)	Cyanosis, respiratory failure	8 m	Dead (8 m)	Cardiomyopathy	CI + II + VI
346	#8	Female	*ECHS1* c.176A>G/c.476A>G	LIMD	(−) ES died at 1 d	Lactic acidosis	0 d	Dead (4 m)	Lactic acidosis, metabolic acidosis	CI
1244	#9	Male	*MPV17* c.451dupC/c.451dupC	HD	(−)	Poor weight gain	1 m	Dead (2 y 9 m)	Hyperbilirubinemia, MTDPS	CI + III + IV
2206	#10	Female	*RARS2* c.944G>C/c.944G>C	NLIMD	(−)	Regression	2 d	Alive (1 y)	Cardiomyopathy, lactic acidosis	NA
1943	#11	Male	*MPV17* c.451dupC/c.308_310del	HD	(−)	RDS	0 d	Alive (3 y)	Hypoglycemia, MTDPS	NA
2307	#12	Male	*BOLA3* c.287A>G/c.287A>G (Maternal UPiD)	CM	(−)	Unconsciousness	4 m	Dead (4 m)	Cardiomyopathy, hypoglycemia, hyperlactatemia	CI + II + VI
2313	#13	Male	*BOLA3* c.287A>G/c.136C>T	CM	(−)	Regression	5 m	Dead (6 m)	Hypotonia, cardiomyopathy	NA

*CM* cardiomyopathy, *LIMD* lethal infantile mitochondrial disease, *NLIMD* nonlethal infantile mitochondrial disease, *LD* Leigh disease, *FCMD* Fukuyama congenital muscular dystrophy, *HD* hepatic disease, *MTDPS* mitochondrial DNA depletion syndrome, *EB* elder brother, *ES* elder sister, *YB* younger brother, *m* month(s), *d* day(s), *y* year(s), *ELBW* infant, extremely low birth weight infant, *RDS* respiratory distress syndrome; Affected complex, affected mitochondrial respiratory chain complex, *NA* not available. *BOLA3*: NM_212552, *ACAD9*: NM_014049, *NDUFAF6*: NM_152416, *TUFM*: NM_003321, *TAZ*: NM_000116, *ECHS*1: NM_004092, *MPV17*: NM_002437, *RARS2*: NM_020320.

Among the 18 pregnancies, 2 resulted in early miscarriage. In the remaining 16 cases that reached the second trimester of pregnancy, 9 opted to have CVS and 6 opted for AC. One case showed possible maternal cell contamination in the CVS sample and subsequently underwent AC. Each causative nuclear gene variant was confirmed by direct Sanger sequencing of PCR-amplified products.

### Index case profiles (Table 1, Fig. 2)

#### Family #1 (Fig. 2a)

Proband (II-1) was a female born at full term (birth weight of 3.10 kg, within the normal range) as the first child to non-consanguineous healthy parents. She exhibited cardiomyopathy with lactic acidosis at 2 months of age, developed heart and liver failure, and died at 5 months of age. Genetic analysis revealed pathogenic variants in *BOLA3* (c.287A>G/c.287A>G); additionally, both parents were heterozygous carriers of this variant.

#### Family #2 (Fig. 2b)

Proband (II-1) was a female born at full term (birth weight of 3.29 kg, within the normal range). She exhibited feeding difficulty at 24 days of age. Laboratory studies revealed severe lactic acidosis, hyperglycemia, and metabolic acidosis; as a result, she was admitted to an intensive care unit. Moreover, she manifested cardiomyopathy, apnea, epilepsy, and liver failure and died of heart failure at 11 months of age. Genetic analysis revealed that she harbored pathogenic variants in *BOLA3* (c.287A>G/c.287A>G), and both parents were heterozygous carriers of this variant.

#### Family #3 (Fig. 2c)

Proband (II-3) was a male born at full term. His elder brother died at 4 months of age, but details were not available. The proband developed lactic acidosis, hyperglycemia, and an elevated creatine kinase level at 17 days of age. He subsequently showed muscle weakness, hypotonia, and cardiomyopathy and died at 17 months of age. Genetic analysis identified pathogenic variants in *BOLA3* (c.287A>G/c.287A>G), and both parents were heterozygous carriers of this variant. Furthermore, he had homozygous variants in *FKTN*. The patient has been reported in a previous study^12^.

#### Family #4 (Fig. 2d)

Proband (II-2) was a female born at full term (birth weight of 2.55 kg, within the normal range). Her elder sister, who had hypotonia with cardiomyopathy, died of acute bronchitis at 6 months of age. The proband was reported to have hypotonia at 5 months of age and exhibited lactic acidosis and hypertrophic cardiomyopathy. Thereafter, she died of acute myocarditis at 13 months of age. She had two different pathogenic variants in *ACAD9* (c.1766-2A>G/c.811T>G), and both parents were carriers of either of these variants.

#### Family #5 (Fig. 2e)

Proband (II-1) was a female born at full term (birth weight of 2.93 kg, within the normal range). Regression and lactic acidosis appeared at 7 months of age. She was diagnosed with Leigh syndrome and died of respiratory failure at 5 years of age. Genetic analysis identified two different pathogenic variants in *NDUFAF6* (c.805C>G/c.226T>C), and both parents were carriers of either of these variants.

#### Family #6 (Fig. 2f)

This family had two probands. Proband (II-1) was a male born at 34 weeks and 2 days (birth weight of 0.81 kg, − 4.4 SD). On the first day, lactic acidosis and apnea were observed, and he died at 8 days of age. Proband (II-2) was the second male child born at 36 weeks and 6 days (birth weight of 1.06 kg; − 5.1 SD). He had congenital heart disease and aortic coarctation and ventricular septal defect. He died of multiple organ failure at 20 days of age. Genetic analysis identified two different pathogenic variants in *TUFM* (c.440T>A/c.162delC), and both parents were carriers of either of these variants.

#### Family #7 (Fig. 2g)

Proband (III-2) was a male born at full term (birth weight of 2.59 kg; within the normal range). Five maternal cousins of his mother (II-5, II-6, II-7, II-8, and II-9) died in the fetal period or during early infancy. Detailed information about them was not available. Hypertrophic cardiomyopathy with cyanosis and respiratory failure occurred in the proband at 8 months of age. Genetic analysis identified a pathogenic variant in *TAZ* (X chromosome-linked gene) (c.367C>T), and his mother was a carrier of this variant.

#### Family #8 (Fig. 2h)

Proband (II-2) was a female born at 39 weeks of gestation (birth weight of 2.94 kg, within the normal range). Her elder sister died of respiratory failure with severe lactic acidosis at 1 day of age. The proband exhibited moderate lactic acidosis and hypotonia shortly after birth. Lactic acidosis was improved by medical treatment; however, she developed severe respiratory and cardiac failure resulting in death at 124 days of age. Metabolic profiling showed no abnormal findings. The brain magnetic resonance imaging at 8 days of age showed T2 high intensities in the cerebral white matter and diffuse brain atrophy at 58 days of age. An autopsy was performed after death. Genetic analysis identified two different pathogenic variants in *ECHS1* (c.176A> G/c.476A>G), and both parents were carriers of either of these variants.

#### Family #9 (Fig. 2i)

Proband (II-1) was a male born at full term (birth weight of 3.2 kg, within the normal range). Poor weight gain and cholestasis were noticed at 1 month of age. The liver showed microvesicular steatosis with swollen hepatocytes and partial necrosis. His jaundice progressed and liver transplantation was performed at 11 months of age. He died of pulmonary hypertension at 2 years and 9 months of age. Genetic analysis identified a pathogenic variant in *MPV17* (c.451dupC/c.451dupC), and both parents were heterozygous carriers of this variant. The proband has been reported in a previous study^13^ (family 18, subject 20).

#### Family #10 (Fig. 2j)

Proband (II-2) was a female born at full term (birth weight of 2.42 kg, within the normal range). She exhibited cardiomyopathy with lactic acidosis at 2 days post-birth. Thereafter, her cardiomyopathy progressed to heart failure, and liver failure occurred. Genetic analysis identified a pathogenic variant in *RARS2* (c.944G>C/c.944G>C), and both parents were heterozygous carriers of this variant.

#### Family #11 (Fig. 2k)

Proband (II-1) was a male born at full term (birth weight of 2.69 kg, within the normal range). He showed tachypnea due to respiratory distress syndrome shortly after birth and was treated with mechanical ventilation for 3 days. Hypotonia and poor weight gain was observed at 3 months of age. Laboratory examinations showed elevated levels of liver transaminases and direct bilirubin. Genetic analysis identified two different pathogenic variants in *MPV17* (c.451dupC/c.308_310del), and both parents were carriers of either of these variants.

#### Family #12 (Fig. 2l)

Proband (II-1) was a male born at full term. He developed impaired consciousness and cardiomyopathy with lactic acidosis at 4 months of age. He died 15 days later. Genetic analysis identified a pathogenic variant in *BOLA3* (c.287A>G/c.287A>G), and his mother was a carrier of this variant.

#### Family #13 (Fig. 2m)

Proband (II-1) was a male born at full term (birth weight of 2.41 kg, within the normal range). Regression appeared at 4 months of age. Additionally, he showed hypotonia and horizontal nystagmus. Symmetrical white matter lesions and lactate peaks were observed in the brain magnetic resonance imaging and magnetic resonance spectroscopy, respectively. Cardiomyopathy had developed and progressed rapidly, and he died at 6 months of age. Genetic analysis identified two different pathogenic variants in *BOLA3* (c.287A>G/c.136C>T), and both parents were carriers of either of these variants.

## Data Availability

The datasets analyzed during the current study are available from the corresponding author on reasonable request.
