# Author Correction: Prenatal diagnosis of severe mitochondrial diseases caused by nuclear gene defects: a study in Japan

**DOI:** 10.1038/s41598-021-02108-2

**Published:** 2021-11-16

**Authors:** Nana Akiyama, Masaru Shimura, Taro Yamazaki, Hiroko Harashima, Takuya Fushimi, Tomoko Tsuruoka, Tomohiro Ebihara, Keiko Ichimoto, Ayako Matsunaga, Megumi Saito-Tsuruoka, Yukiko Yatsuka, Yoshihito Kishita, Masakazu Kohda, Akira Namba, Yoshimasa Kamei, Yasushi Okazaki, Shinji Kosugi, Akira Ohtake, Kei Murayama

**Affiliations:** 1 Center for Medical Genetics, Chiba Children’s Hospital, Chiba, Japan; 2 Department of Medical Genetics/Medical Ethics, Kyoto University School of Public Health, Kyoto, Japan; 3 Department of Metabolism, Chiba Children’s Hospital, 579‑1 Heta‑cho, Midori‑ku, Chiba, 266‑0007 Japan; 4 Department of Pediatrics, Faculty of Medicine, Saitama Medical University, Saitama, Japan; 5 Department of Neonatology, Chiba Children’s Hospital, Chiba, Japan; 6 Department of Clinical Genomics, Faculty of Medicine, Saitama Medical University, Saitama, Japan; 7 Center for Intractable Diseases, Saitama Medical University Hospital, Saitama, Japan; 8 Diagnostics and Therapeutics of Intractable Diseases, Intractable Disease Research Center, Graduate School of Medicine, Juntendo University, Tokyo, Japan; 9 Department of Life Science, Faculty of Science and Engineering, Kindai University, Osaka, Japan; 10 Department of Obstetrics and Gynecology, Saitama Medical University Hospital, Saitama, Japan

Correction to: *Scientific Reports* https://doi.org/10.1038/s41598-021-81015-y, published online 11 February 2021

The original version of this Article contained errors.

In Table 1, the value given for the Status of Family no. #9 was incorrect.

“Dead (2 y 10 m)”

now reads:

“Dead (2 y 9 m)”

In Figure 2, panel G, pro-band I, the square indicating father (3) was incorrectly given as a circle. In addition, the circle indicating mother (4) was omitted. In pro-band IIII, the description under the triangle was incorrectly included.

In panel I, pro-band II, the square indicating male (1) was incorrectly given as a circle.

In panel L, pro-band I, the square indicating the father (1) was incorrectly shown as being a carrier of the pathological variant.

The original Figure 2 and accompanying legend appears below.

**Figure 2:**
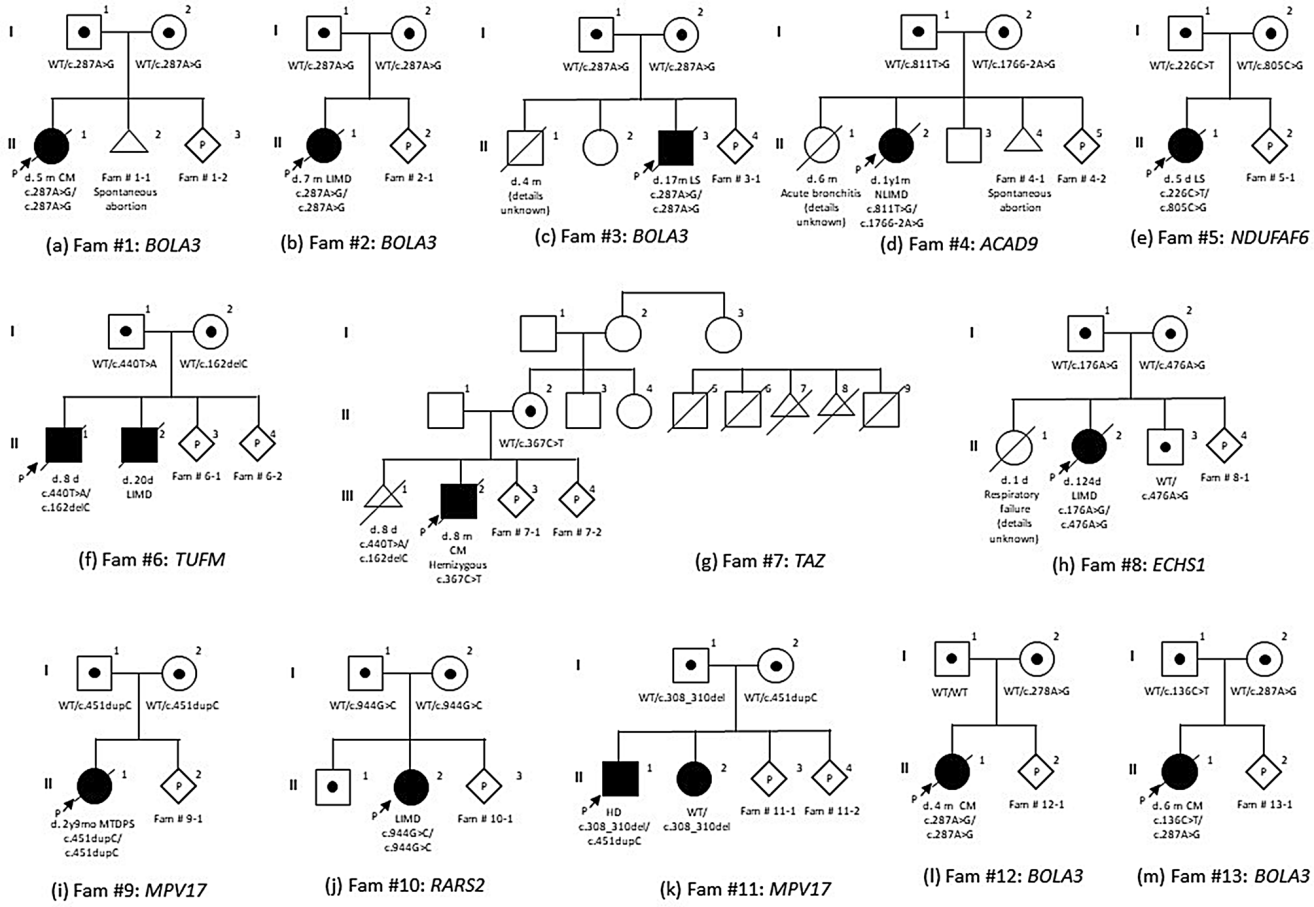
Family tree of 13 probands. One family had X chromosome-linked gene mutation; the other 12 families had autosomal recessive gene mutations. In our cohort, there was no consanguineous couple. *Fam* family, *CM* cardiomyopathy, *LIMD* lethal infantile mitochondrial disease, *NLIMD* non-lethal infantile mitochondrial disease, *LS* Leigh syndrome, *HD* hepatic disease, *MTDPS* mitochondrial DNA depletion syndrome, *WT* wild type, *m* month, *d* day, *y* year.

The original Article has been corrected.

